# The Effect of Photoreactive Diluents on the Properties of a Styrene-Free Vinyl Ester Resin for Cured-In-Place Pipe (CIPP) Technology

**DOI:** 10.3390/ma18102304

**Published:** 2025-05-15

**Authors:** Małgorzata Krasowska, Agnieszka Kowalczyk, Krzysztof Kowalczyk, Rafał Oliwa, Mariusz Oleksy

**Affiliations:** 1 Department of Polymer Composites, Faculty of Chemistry, Rzeszow University of Technology, Al. Powstańców Warszawy 12, 35-959 Rzeszów, Poland; oliwa@prz.edu.pl (R.O.); molek@prz.edu.pl (M.O.); 2 INSTBUD Sp. z o.o., ul. Przemysłowa 3, 32-420 Gdów, Poland; 3 Department of Chemical Organic Technology and Polymeric Materials, Faculty of Chemical Technology and Engineering, West Pomeranian University of Technology, Al. Piastów 17, 70-322 Szczecin, Poland; krzysztof.kowalczyk@zut.edu.pl

**Keywords:** vinyl ester resins, reactive diluents, photopolymerization, CIPP, mechanical strength

## Abstract

Cured-in-place pipe (CIPP) technology is a trenchless rehabilitation method for damaged pipelines in which a resin-saturated liner (often a fiber-reinforced type) is inserted into a host pipe and cured in situ, typically using a UV light beam or steam. This study investigates the influence of selected photoreactive diluents on the photopolymerization process of a styrene-free vinyl ester resin designed for the CIPP applications by evaluating the rheological properties, photopolymerization kinetics (photo-DSC), thermal characteristics (DSC), crosslinking density (gel content), and mechanical properties of thick (15 mm) UV-cured layers. The tested diluents included monofunctional (i.e., methyl methacrylate and vinyl neodecanoate), difunctional (1,6-hexanediol diacrylate, aliphatic urethane acrylates, and an epoxy acrylate), and trifunctional monomers (trimethylolpropane triacrylate, pentaerythritol triacrylate, and trimethylolpropane ethoxylate triacrylate). The key findings demonstrate that the addition of pentaerythritol triacrylate (the most attractive diluent) increases the flexural strength (+6%) and deflection at strength (+28%) at the unchanged flexural modulus value (ca. 2.1 GPa). The difunctional epoxy acrylate caused an even greater increase in the deflection (+52%, at a 5% increase in the flexural strength).

## 1. Introduction

Over the past four decades, cured-in-place pipe (CIPP) technology has served as a critical rehabilitation method for aged underground pipeline infrastructures [1]. This process offers significant advantages in terms of time and cost efficiency (including energy savings). The technique utilizes a composite liner system consisting of a fiber-reinforced sleeve impregnated with thermosetting resins, e.g., vinyl esters, polyesters, or acrylates [2]. The resin formulation represents a crucial material component in CIPP applications [3]. Its curing is achieved via a thermal process (using a steam/hot water circulation system) or a photochemical process using UV/LED light sources mounted on a train system moving at a controlled speed inside the renovated pipe [4].

Vinyl ester (VE) resins represent one of the most commercially significant thermosetting matrix materials for the pipe rehabilitation method. These resins exhibit particularly favorable physical properties for CIPP processes, including competitive costs, low viscosity, and rapid curing kinetics (achievable via thermal, UV, or hybrid methods) [5]. Structurally, VE resins are organic compounds characterized by at least two ester groups (R–COO–R′) with a vinylic functional group (R′). They are typically synthesized by a reaction of (meth)acrylic acid with bisphenol A-based epoxy resins. During the curing process, they typically react with styrene in the presence of radicals generated by peroxide initiators. Similarly to unsaturated polyester resins, styrene serves dual roles, i.e., it is a solvent/diluent that reduces the viscosity of uncured compositions, and it is a chain extender/crosslinker for vinyl ester monomers. Terminal C=C double bonds participate in the crosslinking process through either homopolymerization of the VE resins or copolymerization with the styrene (or other unsaturated monomers) [6].

Photocurable VE-based systems have been tested using various photoinitiators, including bis-acylphosphine oxides, their blends with α-hydroxyketones, and camphorquinone/amine [7]. Photopolymerization rates show significant dependence on the reactivity of the applied reactive diluents; however, studies have been limited to testing methyl acrylate, ethyl acrylate, or butyl acrylate. The mechanical properties of cured VE resin are mainly affected by its prepolymer rigidity, the type and concentration of reactive diluents, and the crosslink density. A higher crosslink density increases the glass transition temperature (T_g_) and modulus while simultaneously reducing the strain-to-failure and impact energy [8].

UV-curable vinyl ester and acrylic resins exhibit excellent mechanical properties, including high chemical and thermal resistance in diverse environments. They are characterized by a high glass mat saturation efficiency and easy processing. Due to these advantages, they are widely employed in the production of glass fiber-reinforced laminates, particularly in the automotive and aerospace industries, as well as in pipeline rehabilitation using trenchless methods [9,10,11]. Despite the benefits of their photopolymerization ability, several undesirable effects may occur during their application: polymerization shrinkage (which depends on the number of unsaturated bonds and monomer functionality), internal stress development, an oxygen inhibition phenomenon, and the presence of unreacted residual monomers, which may be extracted with water or wastewater. A critical parameter of the polymerization process is the final curing degree value, which directly influences the mechanical properties of the prepared inner pipe cover. This parameter depends on multiple factors, such as the type of monomers, reactive diluents, and photoinitiator, as well as the process conditions (e.g., UV intensity, exposure time) [12,13,14,15].

Enhancing the physicochemical and mechanical features of VE resins (employed as binders in UV-cured laminates) presents a significant scientific and technological challenge. Recent investigations have focused on developing novel monomer systems derived from myrcene [16], tung oil [17], flame-retardant compounds [18], and cardanol [19] for VE resin applications. Concurrently, biomaterials engineering research aims to develop high-performance CIPP materials through an advanced composite design. The key requirements for pipeline rehabilitation composites include superior mechanical strength, excellent adhesive properties, and resistance to environmental degradation [20]. These properties are typically achieved using various reinforcing materials (e.g., glass and/or carbon fibers, polyester felt) with unsaturated polyester or VE resin matrices [21].

Generally, the mechanical characteristics of composite structures result from the properties of the resin matrix and reinforcement fiber and fillers, their volume fraction rates, as well as the fiber orientation in the matrix. In light-cured CIPP laminates, the critical processing parameters (including the curing time, UV intensity, radiation source distance, and cured layer thickness) significantly influence the bending behavior of the formed pipe shield. Interestingly, it was revealed that curing at an elevated temperature reduced the flexural strength of laminates [22]. Quality assessment of light-cured composite tubes follows standardized testing protocols [23,24,25], encompassing both short-term evaluations and accelerated aging studies [26]. Long-term performance prediction remains a challenge and requires specialized methodologies to estimate property changes under the operational conditions. These assessments typically focus on creep behavior under sustained loading, cyclic fatigue resistance, and strength reduction factors [27]. Underground pipeline monitoring presents additional difficulties due to limited control of the pipe shield deformation progress and degradation of this system’s sensors in corrosive environments. Recent advances in CIPP material development have demonstrated that multi-walled carbon nanotube-modified composites achieved substantial mechanical improvement in tensile strength: +42% at a concentration of 0.2 wt% of the carbon nanofiller, and +200% at 1 wt% [28,29]. However, this modification requires thermal post-crosslinking as an additional step in the curing process. Environmental concerns regarding leachable compounds (in renovated pipelines) have prompted rigorous investigation [30,31]. Potential contaminants dissipating from partially crosslinked pipe shields include styrene, toluene, and bisphenol A (in epoxy-based systems) [32,33]. Analytical monitoring via ^1^H NMR and GC-MS enables the quantitative assessment of VOC emissions, which initiates optimization of the manufacturing process [34].

This article presents, for the first time, the influence of selected methacrylate, acrylic, and vinyl monomers, which act as photoreactive diluents with different functionalities (mono-, di-, and trifunctional compounds), on the photocrosslinking process of a styrene-free vinyl ester resin (VE). Additionally, this paper presents the thermal and mechanical properties of the resulting photo-cured extra-thick bulk resin layers (15 mm). The investigation employed a commercial styrene-free VE resin, typically used for laminates in the CIPP method for pipeline systems.

## 2. Materials and Methods

### 2.1. Material and Sample Preparation

The following materials were used for the preparation of novel photocurable vinyl ester-reactive diluent (VE-RD) systems:

(a) A styrene-free vinyl ester resin (VE) (L050-LCW-03 FC, AOC GmbH, Kaiserslautern, Germany);
(b) Photoreactive mono-, di-, and trifunctional diluents (RDs):
  - Acrylic monomers: 1,6-hexanediol diacrylate (HDDA; BASF, Ludwigshafen, Germany), aliphatic urethane acrylates (Laromer UA9028 and Laromer UA19T; BASF, Ludwigshafen, Germany), an aromatic epoxy acrylate (Laromer EA9081, BASF, Ludwigshafen, Germany), trimethylolpropane ethoxylate triacrylate (Photomer 4149; IGM Resins, Waalwijk, The Netherlands), trimethylolpropane triacrylate (TMPTA; Allnex, Drogenbos, Belgium), and pentaerythritol triacrylate (PETIA; Allnex, Drogenbos, Belgium);
  - A methacrylic monomer: methyl methacrylate (MMA; Carl Roth, Karlsruhe, Germany);
  - A vinyl monomer: vinyl neodecanoate (VeoVa10; Hexion, Columbus, OH, USA).
(c) A radical photoinitiator: A blend of bis(2,4,6-trimethylbenzoyl)-phenylphosphine oxide (~95 wt%) and ethyl(2,4,6-trimethylbenzoyl)-phenyl phosphinate (~5 wt%) (Omnirad 2100; IGM Resins, Waalwijk, The Netherlands).

The photocurable systems were prepared by mixing VE with the selected unsaturated monomers at a 90:10 weight ratio and the radical photoinitiator (0.25 wt%). All materials were used without further purification. The reference sample contained only VE and Omnirad 2100.

**Figure 1:**
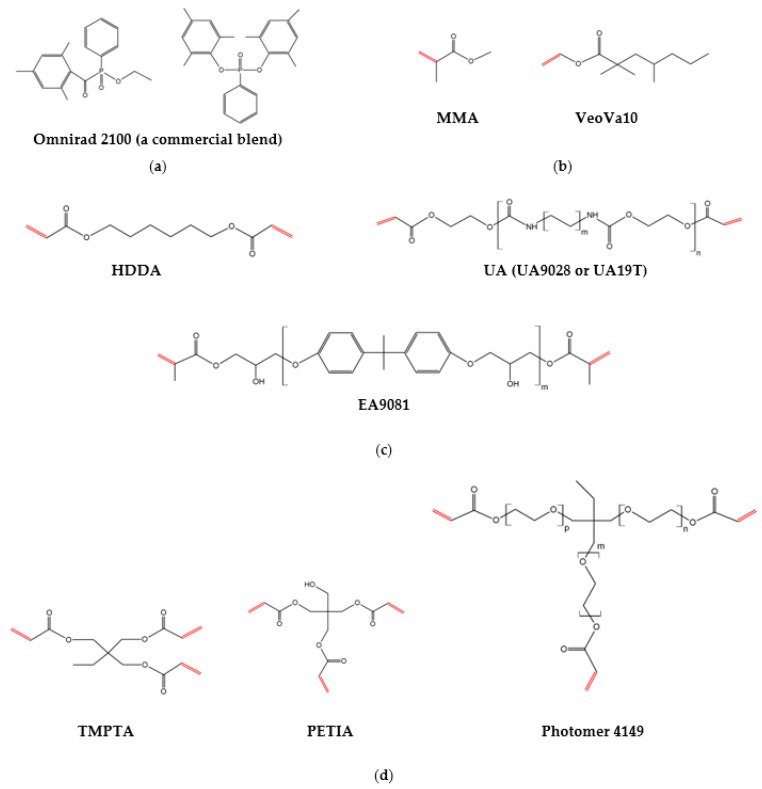
Chemical structures of the radical photoinitiator (**a**) and the photoreactive diluents: monofunctional (**b**), difunctional (**c**), and trifunctional (**d**).

The styrene-free VE resin (L050-LCW-03 FC) is a thixotropic resin, enabling ultra-low VOC emissions during processing and liner installation. It was specially developed for the UV-curing type CIPP technique. The commercial product Laromer EA9081 is labeled by the manufacturer as an epoxy acrylate; however, it is a vinyl ester resin. Although it contains no epoxy groups, the manufacturer’s designation (“epoxy acrylate reactive diluent”) is retained. The diluents were classified by the number of unsaturated groups per molecule (mono-, di-, or trifunctional). The chemical structures of the components are presented in Figure 1.

**Figure 2:**
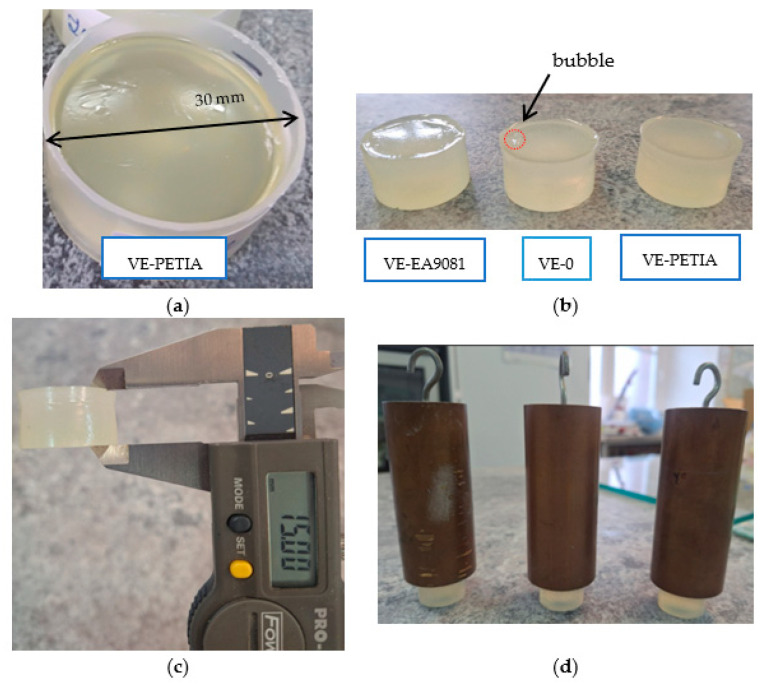
Photos of the UV-cured vinyl ester-reactive diluent compositions (VE-RD): (**a**) in a polypropylene curing jar, (**b**) after their demolding, (**c**) during a dimensional analysis (a thickness measurement), and (**d**) during a mechanical load test (load of 1 kg).

The photocurable vinyl ester-reactive diluent (VE-RD) formulations were cured in polypropylene (PP) jars (diameter of 30 mm, height of 15 mm) under a medium-pressure mercury UV-A lamp (Hönle UV-Technology, Gräfelfing, Germany). The curing process was conducted at an intensity of 8 mW/cm^2^ for 15 min under the ambient atmospheric conditions. UV irradiance was monitored using the Dynachem 500 radiometer (Dynachem Corp., Westville, IL, USA). Two trials were carried out for each system. The cured samples were subsequently characterized through thermal analysis, gel content determination, and visual inspection. Figure 2 presents the macroscopic appearance of the post-polymerization samples.

### 2.2. Methods

#### 2.2.1. Dynamic Viscosity

The viscosity of the uncured the VE-RD systems was measured by means of a DHR-1 dynamic shear rheometer (Discovery Hybrid Rheometer, TA Instruments, New Castle, DE, USA) using rotating spindles according to the EN 13302 standard [35]. The measurements were conducted at 25 °C with a shear rate from 1 1/s to 1000 1/s.

#### 2.2.2. Photo-DSC Measurements

The photocuring kinetics of the VE-RD systems were investigated using the photo-differential scanning calorimetry method (photo-DSC). The measurements were conducted using the DSC Q100 instrument (TA Instruments, New Castle, DE, USA) equipped with UV-photopolymerization accessories. The photo-DSC attachment consisted of two optical fibers: one fiber for irradiation of a sample and the other serving as a reference (irradiation of an empty aluminum pan). The system contained a UV filter (320–390 nm), and the light intensity was 8 mW/cm^2^. All experiments were performed under controlled isothermal (25 °C) conditions in the open aluminum pans (N_2_ purge flow of 20 mL/min). The sample mass was 15.0 ± 0.1 mg. The following kinetic parameters were calculated using the photo-DSC data: the photopolymerization rate (R_p_; W/g), time to the maximum polymerization rate (t_max_; s), and total heat of the polymerization process (ΔH; J/g). The test was triplicated.

#### 2.2.3. DSC Measurements

Thermal transition temperatures for all the obtained UV-cured VE-RD systems were determined by the differential scanning calorimetry method (DSC) using the DSC250 instrument (TA Instruments, New Castle, DE, USA). Measurements were performed under a nitrogen atmosphere with a heating rate of 10 K/min in the temperature range from −80 °C to 250 °C. The glass transition temperatures (T_g_), temperature of onset (T_i_), and maximum temperature (T_max_) of the heat curing reaction, as well as the amount of heat release (ΔH) were determined using the DSC thermographs. The test was duplicated for each sample.

#### 2.2.4. Gel Content

The gel content (i.e., insoluble fractions content; GC) in the UV-cured VE-RD samples was determined via an acetone extraction method. Crushed samples (three samples of each system) were immersed in the solvent at room temperature for 24 h to dissolve the uncured components; then, the solid residues were dried at 60 °C for 24 h and weighed. The gel content was calculated according to Equation (1):

(1) $$GC=\frac{m_{24}}{m_{0}}\;*\;100\;(wt\%)$$

where m_0_ is the mass of a dry sample before immersion (g), and m_24_ is the mass of the dry sample after the immersion and drying (g)

#### 2.2.5. Mechanical Properties

Test specimens for the mechanical characterization of the UV-cured VE-RD systems were prepared in poly (tetrafluoroethylene) (PTFE) molds with dimensions of 80 mm × 10 mm × 4 mm. The liquid UV-photoreactive formulations were poured into the molds and UV-photocured under a UV-A mercury lamp (Hönle UV-Technology, Gräfelfing, Germany) at an irradiance of 8 mW/cm^2^ for 15 min (ambient atmospheric conditions). The UV irradiance was monitored in real time using the Dynachem 500 radiometer (Dynachem Corp., Westville, IL, USA). The flexural properties of the prepared materials were evaluated according to the ISO 178 standard [36] (“Plastics—Determination of flexural properties”) using the Instron 5982 dual-column universal testing machine (Instron, Norwood, MA, USA). The standardized three-point bending test quantified the key mechanical parameters, including the flexural strength, flexural modulus, and deflection at the flexural strength.

## 3. Results and Discussion

### 3.1. Dynamic Viscosity of the VE-RD Composition

**Figure 3:**
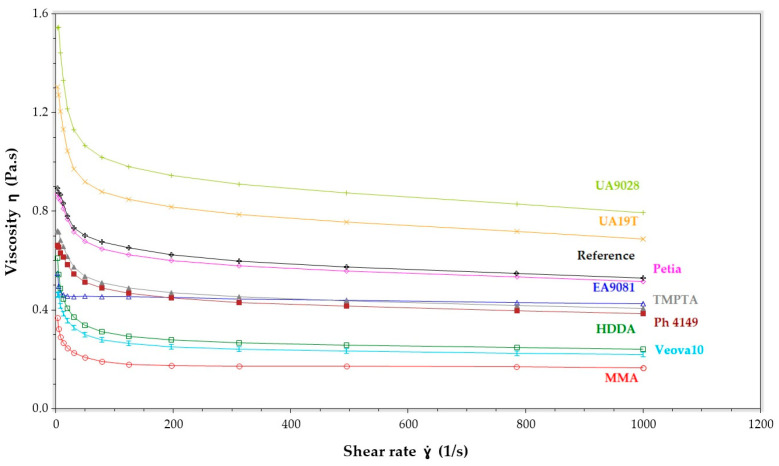
Dynamic viscosity of the UV-curable VE-based compositions with various photoreactive diluents.

Figure 3 illustrates the changes in the dynamic viscosity for VE-based compositions containing the photoreactive diluents. All compositions exhibited a decreasing trend in the relative viscosity as a function of the shear rate, confirming their pseudoplastic (shear-thinning) behavior; all the systems represent non-Newtonian fluids (the viscosity decreases with an increasing shear stress value) [37].

The initial viscosities of the samples at low shear rates (50 rpm) are summarized in Table 1. Compared to the reference sample (VE-0; 700 mPa·s), significantly lower viscosities (210–330 mPa·s) were observed in the compositions containing the monofunctional (MMA, VeoVa 10) or the difunctional monomer (HDDA). These reductions can be attributed to the inherently low viscosity of these reactive diluents.

Decreases in the dynamic viscosity were observed in nearly all the VE-RD compositions, except for the samples with the difunctional urethane acrylates (UA 9028 and UA19T), which exhibited higher viscosities (1070 and 920 mPa·s, respectively). This phenomenon is probably due to the intramolecular hydrogen bonds among urethane groups, a known factor that increases formulation viscosity [38]. Notably, these VE-RD compositions also displayed the most pronounced shear-thinning behavior, suggesting that the applied shear forces disrupted these internal hydrogen bonds.

In contrast, the compositions containing the monofunctional monomers (MMA, VeoVa 10, HDDA) and, unexpectedly, the aromatic-type difunctional epoxy acrylate (EA 9081) demonstrated the greatest resistance to shear-induced viscosity reduction. These rheological differences have direct implications for industrial applications.

The very viscous systems (like UA 9028) may need higher pumping pressures during their processing, but offer advantages in sag resistance in vertical applications. Low-viscosity diluents (e.g., MMA) facilitate good fiber wetting during composite manufacturing; however, the formulation may require modification to prevent drainage. The observed shear-thinning behavior universally enhances processability under shear (e.g., during spraying or injection), while the no-shear viscosity determines static performance parameters, such as resin retention in CIPP liners.

### 3.2. Photopolymerization Kinetics of the UV-Curable VE-RD Systems

**Figure 4:**
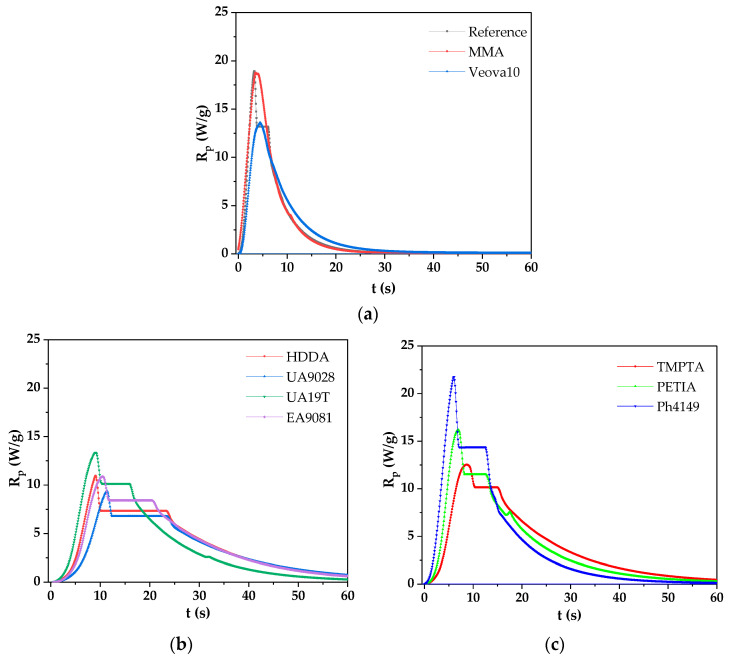
Photopolymerization kinetic profiles of the VE-RD compositions with (**a**) mono-, (**b**) di-, and (**c**) trifunctional reactive diluents.

This study presents, for the first time, the photoinitiated crosslinking of a styrene-free vinyl ester resin (with selected multifunctional photoreactive diluents) using an acylphosphine oxide photoinitiator system. The polymerization kinetic curves (depicted in Figure 4) were analyzed in relation to the diluent functionality. The reference composition (VE-0) contained only the VE resin and the photoinitiator, whose type and concentration were optimized in prior studies to ensure defect-free curing of thick (15 mm) specimens, i.e., to eliminate structural anomalies, such as bulging or shrinkage-induced deformations. Key crosslinking parameters, including time to the maximum reaction rate (t_max_) and total heat evolution (ΔH), are quantified in Table 1.

It is well established in the literature that polymerization processes of multifunctional monomers exhibit several peculiarities distinguishing them from the polymerization of monomers with a single double bond. The most important features include the following: (i) an auto-acceleration phenomenon occurring already at the beginning of polymerization due to a reduction in the mobility of macroradicals within the created polymer network; (ii) autodeceleration observed at later stages of the reaction, when propagation (and termination) becomes significantly controlled by diffusion; (iii) the predominance of so-called “reaction diffusion” as a mechanism controlling termination; (iv) structural heterogeneity of the polymer network resulting from the non-uniform reactivity of functional groups, which leads to the formation of microgel particles [39,40].

The photopolymerization kinetics of the styrene-free vinyl ester resin systems modified with various photoreactive diluents (VE-RDs) reveal significant variations in cure behavior that strongly correlate with both the viscosity and functionality of the diluents. The reference system (VE-0) was characterized by a relatively high viscosity (700 mPa·s). The total amount of heat released after 25 s of its photopolymerization was 233 J/g. Additionally, the reference sample exhibited a characteristic broad polymerization peak with a maximum rate (Rₚ^max^) of ca. 15 W/g, which occurred after 6.6 s. The introduction of the monofunctional diluents (MMA or VeoVe10) significantly reduced the system viscosity and affected the curing behavior differently depending on their molecular structure. The VE-MMA system exhibited the lowest viscosity (210 mPa·s) and accelerated heat release (+16 J/g vs. VE-0), but slightly delayed t_max_ (+0.6 s). This suggests that the low viscosity enhanced mobility and total conversion while the monofunctional nature limited the network formation kinetics. The low viscosity and simple methacrylate structure of the MMA additive significantly accelerated the cure reaction (Rₚ^max^ = 22 W/g after 7.2 s), but VE-MMA showed rapid vitrification and reaction slowdown due to its limited network formation capacity. In contrast, VeoVa10 demonstrated a more sustained reaction profile, with Rₚ^max^ = 18 W/g (at 9 s), owing to its higher molecular weight and branched aliphatic structure, which reduced the vitrification effects, thus maintaining good mobility. VeoVa 10 is a branched vinyl ester monomer with a bulky neodecanoate group, which slows down the polymerization process. Although the tertiary carbon in the ester group can stabilize propagated macroradicals, the overall rate remains moderate due to steric effects [41]. For the systems containing the difunctional photoreactive diluents, the maximum reaction rate values were lower than for the reference sample and the systems containing the monofunctional monomers. In this case (f = 2), the time values required to reach the maximum speed rates were also higher; generally, the photopolymerization processes were longer in relation to the other VE-RD systems. The observed moderate viscosity of the VE-HDDA sample (330 mPa·s) and the flexible alkyl chain structure of this diluent yielded balanced kinetics (t_max_ = 9.0 s), with a slightly increased ΔH (+9 J/g), which indicates efficient network formation. The highly viscous urethane acrylates showed divergent effects: VE-UA9028 (1070 mPa·s) demonstrated both the longest t_max_ (11.4 s) and reduced ΔH (−14 J/g), indicating significant kinetic hindrances from hydrogen bonding and restricted mobility, while VE-UA19T exhibited slightly lower viscosity (920 mPa·s), resulting in a less pronounced inhibition effect. VE-EA9081 stood out among the difunctional systems due to its moderate viscosity (450 mPa·s), the highest ΔH (+17 J/g), and a reasonable t_max_ value (10.8 s). The multifunctional diluents (TMPTA, PETIA, Ph4149) showed the most significant effect on the polymerization kinetics. The VE-Ph4149 system is characterized by extremely rapid cure kinetics (Rₚ^max^ ≈ 23 W/g at 6 s), but the reaction terminated quickly, because the network formation restricted the molecular mobility. This was caused by the chemical structure of this reactive diluent. Photomer 4149 consisted of a trimethylolpropane core with ethoxylate spacers and three terminal acrylate groups. Ethoxylation reduced its viscosity and improved its flexibility compared to the non-ethoxylated TMPTA additive. It is also known that the presence of heteroatoms in a monomer structure (in this case, the presence of an ether background) increases the rate of its photopolymerization process [42,43]. VE-PETIA showed a lower value of the Rₚ^max^ parameter (≈16 W/g) than the reference sample. Additionally, it also displayed complex multi-peak behavior, suggesting a sequential reaction of different acrylate groups or the development of significant microgel regions during the curing process. The high crosslink density achieved with these diluents, however, resulted in increased system viscosity during its curing, which may lead to higher residual stresses in the final material.

### 3.3. Thermal Analysis and Gel Content of UV-Cured VE-RD Compositions

**Figure 5:**
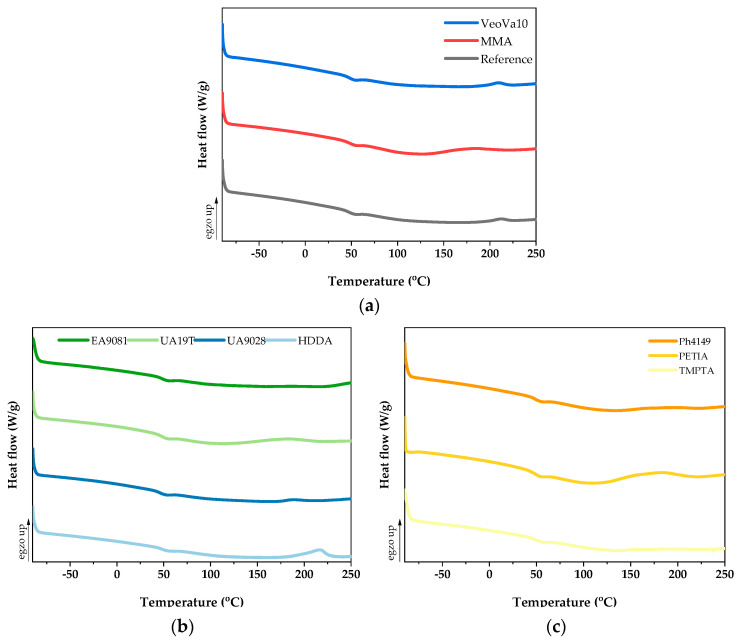
DSC thermograms of UV-crosslinked systems containing (**a**) mono-, (**b**) di-, and (**c**) trifunctional reactive diluents.

DSC thermal analysis of the photocrosslinked vinyl ester resin compositions with different reactive diluents (the VE-RD systems) revealed significant variations in the thermal behavior and crosslinking efficiency, characterized by the onset temperature of a thermal crosslinking process (T_i_), a peak reaction temperature value (T_max_), the released heat value (ΔH), and the gel content (GC). The results are shown in Table 2 and Figure 5. It should be noted that a small bubble was observed inside the reference sample during its photopolymerization (Figure 1b); this defect was absent in all the samples with the photoreactive diluents. This demonstrates that the modification of the basic vinyl ester resin with the diluents yielded beneficial effects. Additionally, all the samples exhibited a slightly sticky surface layer after the photopolymerization process because the UV irradiation was conducted in an air atmosphere and at relatively low UV-light intensity (an oxygen inhibition phenomenon). However, the gel content analysis of the cured compositions indicated that all the polymer systems achieved a high degree of crosslinking (gel content > 99 wt%).

Notably, the highest content of the polymerized fraction was noted for the reference sample and the monofunctional diluent-based systems. This may be a result of their relatively small molecular dimensions and low steric hindrances, which enabled their incorporation into the crosslinked network. The reference sample (VE-0) exhibited high thermal stability; a minimal effect associated with the thermal crosslinking process of the system was registered (the residual heat released was 1.2 J/g). This sample was thermally stable up to 198 °C. In addition, the gel content was very high (99.99 wt%), indicating that the system was fully crosslinked under the applied photopolymerization conditions. An even lower energy effect of the thermal crosslinking process was recorded for the VE-UA9081 sample (only 1 J/g), but it occurred at a slightly lower temperature value in relation to the reference sample (ca. 175 °C). The gel content in this sample was slightly lower (vs. VE-0) (99.97 wt%). Among the photochemically cured VE-RD systems, the sample with the PETIA monomer exhibited the lowest gel content and the highest energy effect of the thermal post-curing process (13 J/g), which began at a relatively low temperature (127 °C). This indicates that the PETIA-modified sample was not fully crosslinked after the realized bulk photopolymerization method. The photo-DSC studies of this system revealed that the photopolymerization process occurred in multiple stages (three distinct peaks are visible in the DSC curves; Figure 4), suggesting the formation of microgels in the system, which resulted in a slightly lower crosslinking density of this sample.

The compositions with either HDDA or UA19T urethane acrylate also exhibited higher energetic effects of the thermal crosslinking reaction (ca. 7 J/g), which correlates with the relatively low gel content values. This phenomenon may be attributed to the low concentration of low-molecular-weight additives in these formulations.

Interestingly, the highest glass transition temperature (50 °C) was observed for the VE-UA19T sample, while the lowest T_g_ (46 °C) was recorded for the UA9028 urethane acrylate-modified material. The systems containing the monofunctional diluents demonstrated similar T_g_ values to the reference sample (48 °C). In contrast, the trifunctional monomer-based materials showed an insignificant T_g_ increment (+1 °C vs. VE-0), resulting from their higher crosslinking density.

### 3.4. Mechanical Properties of UV-Cured VE-RD Compositions

**Figure 6:**
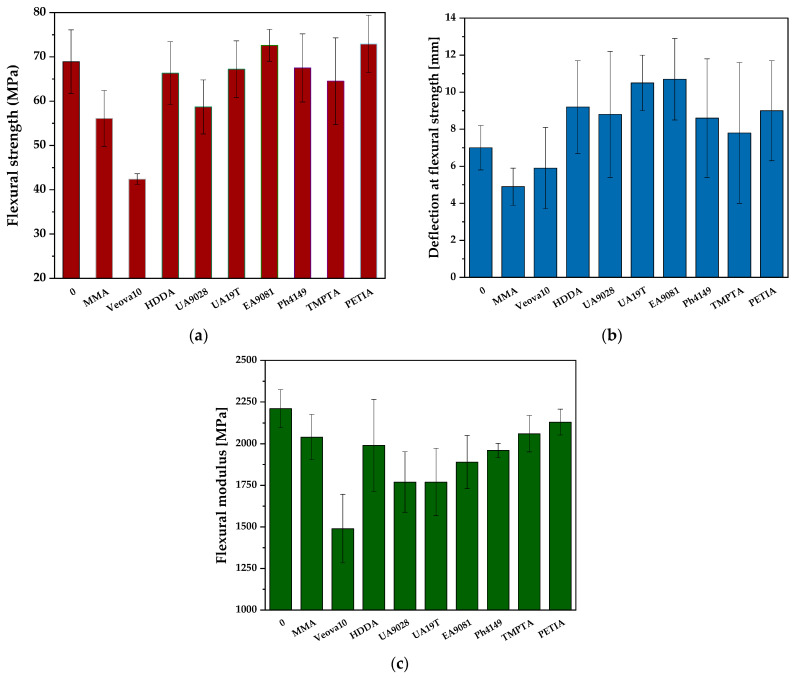
Flexural strength (**a**), deflection at flexural strength (**b**), and flexural modulus (**c**) of UV-cured vinyl ester resin with photoreactive diluents.

The mechanical test results for the UV-cured compositions based on the styrene-free vinyl ester resin and the reactive diluents (with different functionality) are presented in Figure 6. The flexural strength outcomes show significant variations among the tested compositions (Figure 6a). The VE-0, VE-UA19T and VE-Ph4149 samples exhibited quite similar features, while the VE-EA9081 and VE-PETIA materials demonstrated superior performance, with their flexural strength exceeding 70 MPa. Interestingly, only these diluents (EA9081, PETIA) contain a hydroxyl group in their molecules; the mechanical features were likely positively affected by the hydrogen bonds formed in the photo-cured materials [44]. In contrast, the monofunctional reactive diluents (MMA, VeoVa10) exhibited the lowest flexural strength (56.1 and 42.4 MPa, respectively). In the case of the former modifier, its flexural strength correlated with its known limited crosslinking efficiency and brittleness after polymerization [45]. The branched alkyl group in VeoVa10 created steric hindrances that limited its molecular mobility and caused entanglements, which led to reduced rigidity. The difunctional acrylate (HDDA) showed intermediate strength resulting from its linear aliphatic chain, which promoted effective polymerization.

In contrast to the reference sample (VE-0; 7.0 mm, Figure 6b), the di- and trifunctional modifier-based systems exhibited greater deflection at the flexural strength ranging from 8.8 mm (VE-UA9028) to ca. 10.5 mm (VE-UA19T, VE-EA9081) (for f = 2), and from 7.8 mm (VE-TMPTA) to 9.0 mm (VE-PETIA) (f = 3). Generally, all of the difunctional monomers caused superior deflection performance of a thick layer of the UV-cured styrene-free vinyl ester resin. On the other hand, the VE-MMA and VE-VeoVa10 systems showed reduced values of this parameter in relation to the unmodified resin (VE-0), which is attributed to their stiffening influence on polymer networks.

The realized measurements reveal a positive influence of the tested diluents on the flexular modulus of the UV-cured VE resin (Figure 6c). Generally, the modified resin systems were characterized by reduced modulus values, i.e., a greater tendency to deform during the bending process (vs. VE-0). It can be claimed that, with the exception of the MMA and HDDA diluents, the lower the functionality of the modifier, the lower the flexular modulus value of the modified material. Arguably, this relation is caused by different crosslinking densities of the VE-RD systems depending on the functionality of the applied modifiers, as well as their molecular structures and molar concentration in the UV-cured compositions. The lowest modulus value was noted for the sample modified with VeoVa10 (1490 MPa; 2210 MPa for VE-0), i.e., the monofunctional diluent with a relatively long and branched aliphatic chain.

The highest modulus of the modified material was noted for those with TMPTA (2060 MPa) or PETIA (2130 MPa). The trifunctional-type components maximized the crosslinking density and restricted the chain mobility of the VE resin-based matrix (in relation to the other diluents). A relatively high modulus value of the VE-MMA system (2040 MPa) was caused by the above-mentioned brittleness of the polymerized MMA diluent and its low molecular weight (a high molar concentration in the formulation).

## 4. Conclusions

This study investigated the influence of a few photoreactive unsaturated diluents with different functionalities (mono-, di-, and trifunctional) and chemical structures on the rheological behavior and UV-polymerization kinetics of a styrene-free vinyl ester resin designed for the cured-in-place pipe technique (CIPP); additionally, the (thermo)mechanical features of thick (15 mm) UV-cured layers based on the modified resin (VE-RD) were tested.

It was revealed that the addition of monofunctional (MMA and VeoVa10) or difunctional diluents (HDDA) caused the most significant viscosity reduction of the vinyl ester resin (due to their short aliphatic structures). In contrast, the difunctional urethane acrylates (UA19T and UA9028) markedly increased the viscosity of the VE-RD system. Nevertheless, the viscosity values of the VE-RD compositions did not directly correlate with their photopolymerization kinetics. It seems that the chemical structure (mainly the number of unsaturated bonds and heteroatoms) of the diluents played a key role, e.g., the urethane acrylates exhibited relatively slow polymerization rates, whereas the ethoxylated diluent (Ph4149) showed the fastest curing behavior. Under real photopolymerization conditions (an air atmosphere), the thick cured materials were obtained without any structural defects (except for the reference system) and with high crosslinking densities (gel content > 99 wt%). A significant exothermic effect during additional heating of the UV-cured VE-RD system (a DSC analysis) was only observed for the PETIA-modified resin. However, that system (as well as the resin containing a difunctional epoxy acrylate) demonstrated improved flexural strength and greater deflection at the strength in relation to the unmodified sample. Taking into consideration the flexural modulus values, the PETIA-based system achieved the best overall performance.

## Figures and Tables

**Table 1:** Dynamic viscosities of the liquid UV-curable VE-RD systems and their photocrosslinking features.

VE-RD System Symbol	Viscosity (mPa∙s) (25 °C, 50 rpm)	ΔH (J/g)	t_max_ (s)
VE-0	700	233	6.6
VE-MMA (f = 1)	210	249 (+16)	7.2 (+0.6)
VE-VeoVa10 (f = 1)	300	235 (+2)	9.0 (+2.4)
VE-HDDA (f = 2)	330	242 (+9)	9.0 (+2.4)
VE-UA9028 (f = 2)	1070	219 (−14)	11.4 (+4.8)
VE-UA19T (f = 2)	920	231 (−2)	9.0 (+2.4)
VE-EA9081 (f = 2)	450	250 (+17)	10.8 (+4.2)
VE-TMPTA (f = 3)	530	252 (+19)	6.0 (−0.6)
VE-PETIA (f = 3)	640	240 (+7)	6.6 (0)
VE-Ph4149 (f = 3)	510	253 (+20)	6.0 (−0.6)

t_max_—The time to reach the maximum photopolymerization rate of the system; ΔH—the heat released during the photopolymerization of the system; f—functionality of the reactive diluent.

**Table 2:** Thermal analysis results (DSC) and gel content of the UV-cured VE-RD compositions.

Sample	T_g_ (°C)	T_i_ (°C)	T_max_ (°C)	ΔH (J/g)	GC (wt%)
VE-0	48	198	211	1.2	99.99 ± 0.2
VE-MMA	48	140	181	5.9	99.99 ± 0.1
VE-Veova10	49	194	209	1.8	99.99 ± 0.3
VE-HDDA	48	192	216	7.9	99.88 ± 0.2
VE-UA9028	46	175	181	1.0	99.97 ± 0.4
VE-UA19T	50	131	182	7.7	99.95 ± 0.4
VE-EA9081	49	230	265	4.5	99.96 ± 0.2
VE-TMPTA	49	141	185	3.2	99.93 ± 01
VE-PETIA	49	127	180	13.0	99.90 ± 0.2
VE-Ph4149	49	145	187	2.9	99.95 ± 0.2

T_g_—The glass transition temperature, T_i_—temperature at the onset of the thermal curing reaction; T_max_—maximum temperature of the thermal crosslinking reaction; ΔH—the heat of the reaction.

## Data Availability

The original contributions presented in this study are included in the article. Further inquiries can be directed to the corresponding authors.
